# Macromol­ecular phasing using diffraction from multiple crystal forms

**DOI:** 10.1107/S2053273320013650

**Published:** 2021-01-05

**Authors:** Markus Metz, Romain D. Arnal, Wolfgang Brehm, Henry N. Chapman, Andrew J. Morgan, Rick P. Millane

**Affiliations:** a Department of Physics, University of Hamburg, 22761 Hamburg, Germany; b Center for Free-Electron Laser Science, Deutsches Elektronen-Synchrotron (DESY), 22607 Hamburg, Germany; c Computational Imaging Group, Department of Electrical and Computer Engineering, University of Canterbury, Christchurch, New Zealand; d The Hamburg Centre for Ultrafast Imaging, Hamburg, Germany; e ARC Centre of Excellence for Advanced Mol­ecular Imaging, University of Melbourne, Parkville, Australia

**Keywords:** multiple crystal forms, *ab initio* phasing, iterative projection algorithms, X-ray free-electron lasers, XFELs

## Abstract

A phasing algorithm for macromol­ecular crystallography is proposed that utilizes diffraction data from multiple crystal forms – crystals of the same mol­ecule with different unit-cell packings (different unit-cell parameters or space-group symmetries). The approach is based on the method of iterated projections, starting with no initial phase information. The practicality of the method is demonstrated by simulation using known structures that exist in multiple crystal forms, assuming some information on the mol­ecular envelope and positional relationships between the mol­ecules in the different unit cells. With incorporation of new or existing methods for determination of these parameters, the approach has potential as a method for *ab initio* phasing.

## Abstract 2

A phasing algorithm for protein crystallography using diffraction data from multiple crystal forms is proposed. The algorithm is evaluated by simulation, and practical aspects and potential for *ab initio* phasing are discussed.

## 1. Introduction   

Despite the many advances in protein X-ray crystallography, determination of the structures of biological macro­mol­ecules can still be problematic as a result of difficulties in solving the phase problem. Current methods of solution require some kind of initial phase estimates to generate an initial electron-density map.

Experimental methods such as multiple isomorphous replacement (MIR) and multiple anomalous dispersion (MAD) are effective, but they involve additional experimental effort, can be time consuming and expensive, and are not always successful. Problems with MIR include difficulties in obtaining suitable derivative crystals that diffract to sufficient resolution, non-isomorphism between crystals, multiple- and/or low-occupancy heavy-atom sites, radiation damage, scaling of the data, and problems locating the heavy atoms or refining their positions, occupancies or thermal parameters. Complications that can occur with MAD phasing using seleno­methionine (SeMet)-substituted proteins include limited anomalous signal, radiation damage, or a very small or large number of methionine residues, limiting the amount of phase information or making it difficult to determine the heavy-atom substructure.

Mol­ecular replacement (MR) phasing circumvents the need for additional experiments, but despite the power and widespread application of this approach, MR is not always suitable or effective. An homologous mol­ecule of known structure may not be available to provide initial phase estimates. The target mol­ecule may contain new structural motifs and/or the sequence homology may not be reflected in structural homology. The MR phases obtained may not be accurate enough to produce an interpretable map, or they may be accurate only at insufficient resolution. It may not be possible to position the model accurately enough in the target unit cell to obtain accurate enough phases. Model bias can occur when the measured intensities from the target are insufficient to pull the phases away from those of the model, with the result that the solution may resemble, or contain aspects of, the model structure. Various strategies are available to help reduce model bias, such as SIGMAA weighting (Read, 1986 ▸, 1997 ▸), omit maps (Hodel *et al.*, 1992 ▸), optimal phase combination (Cowtan, 1999 ▸) and the prime-and-switch method (Terwilliger, 2004 ▸). However, as the proportion of the target structure represented by the MR model decreases, the MR phases can become incapable of generating an interpretable electron-density map. Finally, since the model must be structurally similar to the target mol­ecule, the method tends to be ineffective in cases where the target contains new, currently unknown, folds. MR can therefore be unsuitable for discovering new, novel structures.

As a result of the experimental burden and potential difficulties associated with current methods, there is still an active interest in developing new phasing methods to complement current techniques. In particular, there is interest, and potential value, in the development of new phasing methods that require little, or no, initial phase information. Methods requiring no initial phase information are often referred to as *ab initio* phasing. While *ab initio* phasing is a worthy goal, in practice, some limited additional information will usually be required to solve the phase problem. The key consideration is the amount of information required, and the ease with which it can be obtained and incorporated into a phase retrieval algorithm.

In this paper we describe an approach to phasing in protein crystallography that utilizes diffraction data collected from the same mol­ecule in different crystal forms. In some cases, diffraction data from multiple crystal forms may be readily obtained, during crystallization screening for example, and be more convenient than using alternative experimental phasing techniques. In fact, it is not uncommon for proteins to crystallize in more than one crystal form, but the additional crystal form(s) are often not reported, because the best crystal is chosen for structure determination. There is potential advantage, however, in not discarding data from the other crystal form(s), but using them as supplementary data for phase determination. Such an approach, which uses only diffraction data from crystal forms containing sufficiently isomorphous mol­ecules, does not depend on a model structure. It is therefore not subject to model bias. Since neither experimental phases nor a known structure are needed, the approach can be considered a form of *ab initio* phasing, depending on what other information is required. The objective is to use little, or no, information in addition to the diffraction data, to work towards a method for *ab initio* phasing. Successful application of such an approach could significantly reduce the experimental demands of structure determination in some cases.

The idea of using diffraction measurements from the same mol­ecule crystallized in two or more crystal forms for structure determination is not new. Sayre (1952 ▸) recognized that if the mol­ecular transform could be measured at positions between the reciprocal-lattice points, then additional information is obtained that would constrain the phases. This is, as we shall see, pertinent to the problem at hand. Bragg & Perutz (1952 ▸) proposed that making additional measurements by shrinking or expanding the crystal lattice constants would provide additional information to assist in phasing. They made diffraction measurements of haemoglobin at different shrinkage stages, and were thus able to estimate the mol­ecular transform amplitude at different positions in reciprocal space. Tracking these amplitudes in centric zones showed where the transform passes through zero, giving the position of the sign changes, allowing the signs (phases) of the structure factors in these zones to be determined.

Rossmann & Blow (1962 ▸, 1963 ▸) recognized that, in the presence of non-crystallographic symmetry (NCS), one can enforce equivalence of the electron density in the subunits (or mol­ecules), giving additional information that should help in structure determination. They also recognized that the same idea applies in the case of the same mol­ecule in different crystal lattices, *i.e.* one can enforce equality of the density of the equivalent mol­ecules in the different unit cells. The presence of NCS, or different crystal lattices, effectively provides information between the reciprocal-lattice points, thus emulating the situation described by Sayre. Applying identity of the electron density of the subunits, or mol­ecules, gives equations that express relationships between the structure factors which can, at least in principle, be solved for the phases (Main & Rossmann, 1966 ▸; Crowther, 1967 ▸). However, solution of the equations is unwieldy, and this approach was never used for phase determination.

A breakthrough in the application of these ideas occurred when the problem was reformulated in real space, leading to a solution based on averaging of the electron density over the copies of the subunits or mol­ecules, as part of an iterative scheme (Buehner *et al.*, 1973 ▸; Bricogne, 1974 ▸). This was the forerunner to modern electron-density modification, which was, and is, used to much advantage for phase refinement and extension, from an initial experimental phase set, using constraints such as NCS and solvent regions. It is worth noting that what is now generally meant by MR, in which the *identical* mol­ecules (or subunits) as described above are replaced by *similar* mol­ecules, and the phases of one (the known model structure) are used as approximate phases for the other (the unknown or target structure), grew out of this early work.

Although the primary use of electron-density averaging has been in using NCS to improve phases, averaging between different crystal forms has also been used for the same purpose, often referred to as multi-crystal, or cross-crystal, averaging. Some examples are as follows. Multi-crystal averaging has been used to improve MAD phases (Brejc *et al.*, 2001 ▸) and MR phases (Li *et al.*, 2004 ▸; Lomakin *et al.*, 2007 ▸). Simonović & Steitz (2008 ▸) used cross-crystal averaging to resolve apparent differences between two ribosome structures. Cross-crystal averaging has also been used to help solve structures with data suffering from heavy-atom non-isomorphism and multiple SeMet sites (Busby *et al.*, 2016 ▸), as well as twinning and pseudo-symmetry (Bunker, 2016 ▸). In an application that is close to *ab initio* phasing, Yoshimura *et al.* (2016 ▸) solved the structure of a viral capsid domain using cross-crystal averaging and an envelope derived from the ill-defined density in the domain region of the complete virus. Li & Li (2011 ▸) describe an interesting application of cross-crystal averaging in which they reduced model bias in an MR solution by averaging between a known part of the target structure and the search model in its own crystal form. However, despite these various applications, multi-crystal averaging has so far been used, almost exclusively, for refining phases obtained using another technique, as part of a conventional density-modification scheme, rather than for *ab initio* phasing.

In this paper we revisit the idea of using diffraction data from the same mol­ecule crystallized in more than one unit cell, with the objective of developing a method for *ab initio* phasing. We approach the problem by developing a method based on iterative projections, that can converge to a solution starting from a random phase set.

The problem is formulated in the next section and conditions under which a unique solution is expected are examined in Section 3. The main tool that we use, iterative projection algorithms, are briefly reviewed in Section 4. Our reconstruction algorithm is described in Section 5. Some preliminary simulations that show the potential of this approach are presented in Section 6, and concluding remarks are made in Section 7.

## 2. Problem formulation   

The approach described here depends, of course, on the mol­ecules in the different crystal forms being isomorphous, or at least isomorphous to a major degree. Although this is often the case, there are also cases where the different crystallization conditions and different chemical environment of the proteins lead to larger structural changes (see, for example, Betts *et al.*, 1994 ▸; Carter *et al.*, 1994 ▸). If these changes, or the presence of different ligands, are large enough, then the method we propose may not be effective. For the purposes of formulating the problem, we assume here that the mol­ecules in the different crystal forms are identical.

Consider a mol­ecule, or mol­ecular assembly, with electron density 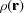, that packs into *N* different crystal forms with various unit-cell dimensions and possibly different space-group symmetries. We perform diffraction experiments on each crystal form and extract a set of integrated Bragg intensities 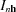, where 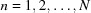 indexes the crystal forms and 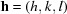 are the Miller indices of the Bragg reflections. Each crystal form is made up of copies of the same unit 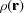, where the relative position and orientation between each of the units are different for each crystal. We refer to this unit simply as the ‘mol­ecule,’ although 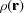 might consist of a number of mol­ecules.

We note that *different crystal forms* will generally correspond, in practice, to different cell constants and/or space groups. The case of identical cell constants and space group, but different packings of the mol­ecules within the different crystals, is also covered by our formalism, but is unlikely to occur in practice. This case is discussed further, later in this section.

We now describe the measured quantities 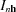 in terms of the common mol­ecule 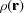, the object of interest. The diffraction intensities 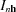 are the squared amplitudes of the structure factors 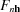 so that
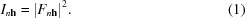
It is convenient to describe the relationship between 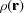 and 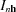 using the symmetry operators within each crystal and the geometrical relationships between the different crystals. Denoting the density in one unit cell of crystal *n* by 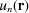, then
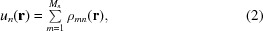
where 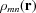 is the density of the *m*th asymmetric unit in the *n*th crystal, and there are 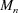 asymmetric units in the unit cell of crystal *n*.

Let the rotation matrix 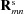 and translation vector 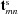 be the space-group symmetry operators for the *m*th asymmetric unit in crystal *n*, relative to asymmetric unit 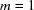, so that
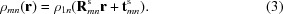



The geometrical relationship between the mol­ecules in the different crystal forms is described by inter­crystal position (rotation and translation) operators (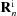, 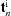), that relate the first (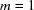) asymmetric unit in each crystal to that in crystal 1, *i.e.*

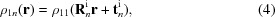
and we arbitrarily choose the first mol­ecule (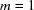) in the first crystal (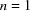) as the reference density for the mol­ecule, *i.e.* 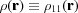.

We now define the global position operators (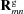, 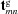) that relate the position of 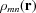 to that of 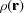, *i.e.*

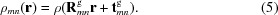
It is then easily seen that the global operators are given in terms of the space group and inter­crystal operators by
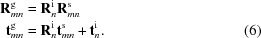



The structure factors 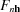 for crystal *n* are equal to the continuous Fourier transform of the contents of the *n*th unit cell 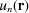, sampled on the reciprocal lattice for that crystal form. The reciprocal-lattice points for the *n*th crystal are denoted 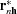. Let 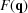 be the continuous Fourier transform of the mol­ecule 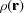, where 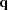 is the continuous position variable in reciprocal space, *i.e.* 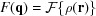, where 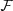 denotes the Fourier transform operation. The structure factors for crystal form *n* are then given by
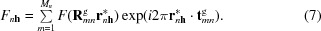
With reference to (7), it is informative to consider the following. Since the reciprocal lattice possesses the rotational symmetry of the direct-space lattice, for a single crystal form *n*, the points 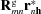 in (7) are points on the same sampling lattice for different *m, i.e.* 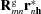 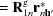 for some 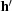, for any *m*. Therefore, for a single crystal form, the intensity measurements 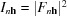 contain information on the continuous transform amplitude 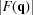 only on the corresponding reciprocal lattice at 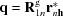. Increasing the number of asymmetric units in the unit cell (*i.e.* higher crystal symmetry) therefore does not increase the sampling of the continuous transform of the mol­ecule 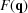.

For the case of multiple crystal forms however, the sampling lattices for 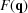 in (7) for the different crystals are necessarily distinct, *i.e.* 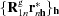 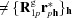 for any 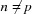. Equation (7) then shows that data from multiple crystal forms provide information on different samplings of different linear combinations of the continuous transform of the mol­ecule 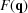. The undersampling of 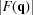 by the Bragg samples for a single crystal form is then reduced, and each additional crystal form with a different unit cell provides additional (independent) information on 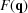.

We note that it is theoretically possible that *individual* sample points for two crystal forms could coincide, *i.e.* 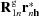 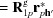 for some 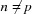, and some 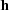 and 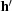. In this case, the different sets of phase factors in (7) result in different linear combinations of the samples of the continuous transform for the different crystals, so giving independent information on 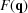 in this case also.

It is worth considering the following. In the different crystal forms referred to here, the mol­ecules adopt different crystal packing arrangements, and it is these different geometrical relationships between the mol­ecules in the different crystals that result in the different samplings of the mol­ecular transform amplitude. It is not in fact *necessary* (as alluded to above) that the unit-cell constants, or the space group, are different. If both the unit-cell constants and space-group symmetries were identical for the different crystals, but the intercrystal position operators are distinct, then the global position operators are different for each crystal, and the data from the different crystal forms are independent via (7). If only the rotational intercrystal operators were different, then the result is a different sampling of reciprocal space for each crystal. If only the translational intercrystal operators were different, then the sampling of the mol­ecular transform is actually the same, but the phase factors in (7) result in measurements that are different, independent, linear combinations of the amplitudes at the sample points. These observations emphasize that it is the different packing arrangements of the mol­ecules, rather than necessarily different unit cells, that ensure there are additional independent Bragg data.

The situation described in the previous paragraph can be related to a recent proposal for using diffuse diffraction from imperfect (disordered) crystals for structure determination (Ayyer *et al.*, 2016 ▸; Morgan *et al.*, 2019 ▸). In that case, there is a single set of cell constants and space group, but the mol­ecules are subject to small random translations from one unit cell (or asymmetric unit) to the next, within a single crystal. The resultant different local packings give rise to diffuse diffraction that contains information additional to that contained in the Bragg reflections. While different to the case considered here, since in that case there are translations *within*, rather than *between*, crystals, there is a fundamental link between the two situations.

With the setup as described above, the problem at hand is to reconstruct the mol­ecule 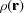 from the diffraction data 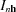 for a number of crystal forms *n*. We consider the *ab initio* case where there is no initial phase information, although we do assume that some limited real-space information is available.

## 3. Uniqueness   

Our objective is reconstruction of the electron density from the diffraction amplitudes (or intensities) from multiple crystal forms alone, without other phase information. This is a form of *ab initio* phasing, and an immediate question of fundamental importance is, do the diffraction data provide sufficient information for a unique solution to the phase problem in the absence of other information?

A simple view of the problem is as follows (Millane & Arnal, 2015 ▸). Since the structure factors (amplitudes and phases) provide just enough information to determine the electron density, and since the amplitudes and phases are independent, then loss of the phases represents loss of half of the required information. The Nyquist spacing for the intensity 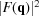 is half the reciprocal-lattice spacing in each direction (Millane, 1990 ▸), so that the Bragg samples are below the Nyquist density. This means that the intensities on a second reciprocal lattice as provided by a second crystal form (as described in the previous section) provide information that is independent of that from the first crystal form. Therefore, data from a second crystal form should be just sufficient to make up for the lost phase information. Reconstruction from the amplitudes only is a nonlinear problem however, so that these data will not quite be sufficient, but data from a third crystal form should provide sufficient information for a unique solution. If other *a priori* information is available, such as known solvent regions, for example, then data from two crystal forms may be sufficient (or even a single crystal form if the solvent fraction is large enough). Of course, data from more crystal forms would be desirable in practice to help compensate for the effects of noise, missing data *etc*.

The above description can be presented in a more formal way as follows. Uniqueness properties of the phase problem for single objects (not necessarily crystals) are conveniently characterized via the constraint ratio Ω (Elser & Millane, 2008 ▸), that describes the number of available independent diffraction amplitude data relative to the number of parameters describing the electron density. Uniqueness requires that 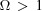, *i.e.* the number of independent data exceeds the number of parameters. As a result of errors in the data and other uncertainties, a margin will be needed in practice, and a value somewhat greater than unity is required. Various results indicate that Ω greater than about 1.5 might be required in practice (Millane & Lo, 2013 ▸).

In the crystallographic case, where the number of data are limited by the Bragg sampling, 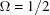, or 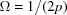 if it is known that the protein occupies a volume fraction *p* of the unit cell (Millane & Arnal, 2015 ▸). Therefore, with data from a single crystal form, the problem is highly underdetermined.

For diffraction data collected from the same mol­ecule, crystallized in *N* different crystal forms, the total number of data is equal to the sum of the number of unique Bragg data from each crystal form, but the number of parameters describing the density is the same as for one crystal form. The data-to-parameter ratio, which we denote Ξ, is then given by
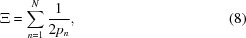
where 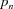 denotes the protein content of crystal *n*. If the protein contents are similar, *i.e.* 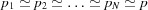, then (8) reduces to
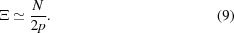
Although the protein contents may or may not be similar in particular cases (Chruszcz *et al.*, 2008 ▸), we consider, for simplicity, the case of identical protein contents in the following analysis. This does not affect the conclusions in any material way. Inspection of (9) shows the somewhat obvious result that the data-to-parameter ratio increases linearly with the number of crystal forms. Consequently, for example, even with a low solvent content of say 30% (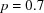), for two crystal forms 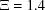, and for three crystal forms 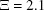.

We now consider the relationship between the simple data-to-parameter ratio Ξ and the more informative constraint ratio Ω. Although Ξ increases without bound as *N* increases, the information content of the data does not increase indefinitely. The maximum amount of information available occurs for the case of a single mol­ecule, where one measures the continuous diffracted intensity and, as a result of the sampling theorem, the constraint ratio for a single object is fixed and bounded for a particular object shape. For a compact (convex and centrosymmetric) single object, 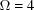 (Elser & Millane, 2008 ▸). Although low-resolution mol­ecular envelopes are not exactly compact in this sense, they are generally approximately so, and 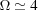 is a reasonable approximation in most cases. Bragg sampling can only reduce the value of Ω, even for multiple crystals. Since, as argued above, the diffraction data from each crystal form are expected to be independent, but Ω cannot exceed 4, we conclude that
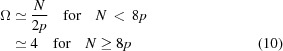
and for the conservative case of no envelope (protein content) information (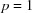)
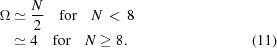



The constraint ratio as described in the previous paragraph assumes that the sampling patterns are not structured in specific ways. For example, if two cell constants changed and one remained unchanged, then the continuous intensity is undersampled along one direction and Ω would be reduced by a factor 2. The implications of this are that, in such cases, the reconstruction problem is likely to be more difficult and more noise-sensitive. As a matter of interest, such a situation is related to the case of 1D and 2D crystals (Millane, 2017 ▸; Arnal & Millane, 2017 ▸).

Inspection of (10) and (11) shows that data from three or more crystal forms should be sufficient in general for a unique solution in the absence of mol­ecular envelope information, and that two crystal forms should be sufficient if there is some envelope information, since in these cases 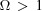. In practice, the problem is better determined if data from more crystal forms are available, since the additional data help to ameliorate the effects of errors. The equations also suggest that there are diminishing returns for more than eight crystal forms, since the constraint ratio saturates in this case. However, in practice, data from additional crystal forms will be beneficial. Excess data correspond to *oversampling* the continuous transform, and it is known that oversampling by a factor *f* relative to the Nyquist spacing reduces the effect of the noise level in the sample values by a factor *f*, and also compensates for the effects of missing samples (Marks, 1983 ▸, 1991 ▸). In practice then, data from more crystal forms will always be beneficial. These results are in accord with the simple analysis described above.

The constraint ratio defined by (10) and (11) assumes that the data from each crystal form are all independent. It is worth considering further the implications of two reciprocal-lattice points from two crystal forms being either too close together or being too widely separated (due to, for example, one or two of the unit-cell dimensions being quite similar, as described above). It is known that *bunching* or *gaps* in a sampling sequence increases the sensitivity of inter­polation to errors in the sample values (Yen, 1956 ▸; Marks, 1991 ▸). In this regard, it is instructive to consider two cases that can arise: the first when the cell constants are quite different, and the second when they are quite similar.

If the unit cells are quite different (in which case the space groups are also likely to be different), then the reciprocal lattices are also quite different. The reciprocal-lattice points are then generally unlikely to be close together. In the event that two reciprocal-lattice points are close together, then, as a result of the different global position operators, the structure-factor amplitudes will still be independent, as described in Section 2. Therefore, for unit cells that are quite different, the data from the different crystal forms will generally be independent.

If, on the other hand, the different unit cells are similar, then the reciprocal lattices will also be similar, and there is a greater likelihood that some reciprocal-lattice points will be close together. For similar unit cells, the global position operators 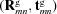 are also likely to be similar, reducing the independence of data at reciprocal-lattice points that are close together. This effect can be assessed as follows. The Nyquist spacing for the continuous diffracted amplitude (or intensity) is half the reciprocal-lattice spacing. Consider initially a 1D lattice with spacing (cell constant) *a*. The Nyquist spacing for the diffracted amplitude is then 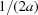. Now consider a second unit cell with cell constant that is larger by δ, *i.e.* cell constant 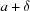. It is easily seen that the distance between two corresponding reciprocal-lattice points from the two lattices is small at low resolution and increases with increasing resolution. At resolution *d*, for small δ, these low-resolution reciprocal-lattice points are a distance of approximately 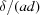 apart. It is convenient to express this distance as a fraction Δ of the Nyquist spacing 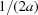, so that 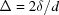. We show in Appendix *A* [App appa] that if two samples are a fraction Δ of the Nyquist spacing apart, then the noise in those sample values is amplified by a factor 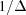. For example, if we consider noise amplification to be significant for 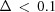 (as considered in the Appendix[App appa]), and for a difference in unit-cell constant δ = 1 Å, using the above equation shows that noise amplification would become significant only at resolutions *d* less than 20 Å. This resolution threshold will be lower for larger differences in the unit-cell dimensions.

Consider, for example, two crystal forms in which one cell constant differs by 1 Å and the other two cell constants differ by larger values. The noise sensitivity is dominated by the cell constant with the smaller difference and, following the above analysis, noise amplification will be significant out to a resolution of 20 Å along the corresponding direction in reciprocal space and to high resolution in the other two directions. If the maximum resolution of the data is 2 Å, then these data represent approximately 15% of the total data set. Since 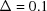 at 20 Å resolution, the average value of Δ is 0.05 over the low-resolution data. The overall noise amplification factor is then (0.15/0.05 + 0.85/1.0), *i.e.* the noise is amplified by a factor of about four. This example illustrates the noise sensitivity in the case of unit cells with similar dimensions, and the need for either data from more crystal forms in that case, or larger differences in the cell dimensions.

Another factor that could potentially affect uniqueness is overlap between the mol­ecular envelopes of the asymmetric units in a single unit cell. Envelope overlap is discussed in more detail in Section 5. The problem considered is constrained as a result of the equivalence between the electron densities of the mol­ecules in different unit cells. In overlap regions, the electron densities of the individual mol­ecules may not be available, limiting application of this equivalence. However, the relationship between overlap and uniqueness is complicated, and depends on the details of the nature of, and relationships between, the overlap regions in each unit cell, in a particular case. Since the mol­ecules themselves do not overlap, the size of overlap regions will be small. The worst-case situation would occur when only the overlap-free region of the envelope provides a constraint between the electron densities. For two crystal forms, for a small degree of overlap, this would reduce Ω by a factor of approximately 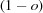, where *o* is the fraction of the envelope volume subject to overlap. For 5% overlap, for example, this reduces Ω by a factor of only 0.95. For more crystal forms, the effect will be less significant since Ω is larger. We conclude, therefore, that envelope overlap will have minimal effect on uniqueness of the reconstruction problem.

Finally, we consider the effect of non-equivalence of the mol­ecular structures in the different forms on uniqueness. As noted above, the structures of the mol­ecules in different crystal environments will generally be slightly different, and so the data from the different crystals will correspond to slightly different electron densities. Phase determination against the combined crystal form data sets is expected to produce, at best, an electron-density map that approximates the average of the densities from the individual crystal forms. This average map would be useful when multiple, slightly different, conformations exist. In some cases, structural differences as a result of different packings will tend to be concentrated near the periphery of the mol­ecule, as a result of different inter­mol­ecular contacts between surface loops. The resolution of the reconstruction may then be higher in the interior of the mol­ecule than near the edges, allowing chain tracing in the interior, followed by model-building and refinement to improve the phases and the map on the periphery. As noted above, in cases where there are large differences between the structures in the different crystal forms, our approach may not be applicable.

In summary then, the data will be independent in most cases, and the problem posed is expected to have a unique solution with data from three or more crystal forms, and from two crystal forms in favourable cases, although it is likely that some additional information on the object, such as a molecular envelope, will be needed. Additional crystal forms will be beneficial in terms of ameliorating the effects of noise, errors *etc*. Complicating factors such as similar unit cells, envelope overlap and small differences between the mol­ecular structures may affect uniqueness, but only to a manageable degree.

## 4. Iterative projection algorithms   

We use the approach of constraint satisfaction, or iterative projection algorithms (IPAs), to solve the reconstruction problem. IPAs are an effective and frequently used type of algorithm for *ab initio* phase retrieval, and other reconstruction problems, for example in crystallography and other areas of imaging. The basis of this approach is outlined here and the reader is referred elsewhere for more details (Elser, 2003 ▸; Marchesini, 2007 ▸; Millane & Lo, 2013 ▸).

Values of the electron density of the object (mol­ecule) on a sampling grid are represented as points in a high-dimensional vector space, one dimension for each sample, with each coordinate value representing the value of the object at that sample. The data (diffraction intensities in our case) are represented by a manifold (set) in the vector space, *i.e.* the manifold contains all points that represent objects which would produce the given data. Since the data are usually incomplete, *i.e.* insufficient by themselves to define the object, the data manifold is high-dimensional. Other information about the object (generally in real space) is represented by another manifold in the vector space, *i.e.* this manifold contains the points representing all objects that are consistent with all the real-space information, or constraints. This manifold is also generally high-dimensional. A valid solution is one that satisfies both the data and object constraints, and therefore lies in the inter­section of the two manifolds. If the problem is well determined, or has a close-to-unique solution, this inter­section will be *small* in extent, *i.e.* close to a point. The reconstruction problem then is to find a point in this inter­section.

IPAs are algorithms used to find a solution, or a point in the inter­section of the constraint sets. They start at a random point in the vector space, *i.e.* a random object, and perform a sequence of moves, or updates, to this point, or object, with the objective of locating a point in the inter­section. IPAs have three components: constraint sets, projections and an update rule. Constraint sets represent objects that satisfy either of the diffraction data or real-space constraints, as described above.

A projection is an operation that takes an object to the closest (in the Euclidean distance, or squared difference, sense) object in a constraint set. The new object then satisfies the constraint. For most IPAs, and the ones we consider here, there are two constraint sets and two projection operators. In our case, one projection operator projects onto the real (or object)-space constraints and the other onto the reciprocal-space (or Fourier amplitude, or data) constraints. We denote these two projection operators by 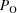 and 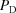, respectively.

The final component in an IPA is the *update rule*. The update rule is the operation that takes the current object estimate, or the *iterate*, to the next estimate. One such step is called one iteration of the algorithm. The update rule is a combination of the two projection operators. Different IPAs have different update rules, and may have different kinds of convergence behaviour.

The error reduction (ER) algorithm is the simplest IPA. It projects alternately onto the object and data constraint sets. The update rule for the *l*th iterate, 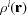, is then given by
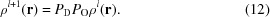
Each iteration of the ER algorithm reduces the summed distance to the two constraint sets, but the algorithm converges to a *local* minimum that usually does not satisfy both constraints. Thus the ER algorithm is a local minimizer. ER corresponds to the usual forms of conventional electron-density modification in protein crystallography (Millane, 1990 ▸; Millane & Lo, 2013 ▸).

The main IPA that we use here is the difference map (DM) algorithm (Elser, 2003 ▸), which has better global convergence behaviour than the ER algorithm. This is particularly important in the case at hand, for *ab initio* phasing, where we do not start with initial phase estimates or an approximate solution. The DM algorithm uses so-called *relaxed* projections. Relaxed projections do not project to the closest point in the constraint set, but ‘under- or over-shoot’ this point. The relaxed projection *T* is given by
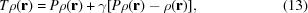
where *P* stands for any projection operator, either the data or object projection 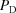 or 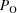. The relaxation parameter γ controls the amount of relaxation, and may be positive or negative. The update rule for the DM algorithm is given by (Elser, 2003 ▸)
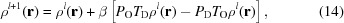
with 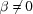, set usually to 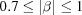, and the relaxation parameters for 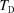 and 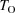 usually set to 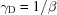 and 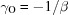 (Elser, 2003 ▸). There is then a single parameter β which is effectively a step size. For the DM algorithm, the iterate itself is not an estimate of the solution, but an estimate of the solution at convergence of the algorithm is given by 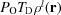 or 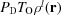 (Elser, 2003 ▸).

## 5. Reconstruction algorithm   

Our objective is to reconstruct the mol­ecular electron density 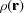 from the diffraction data 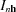 for the *N* crystal forms. Our interest here is with *ab initio* phasing, *i.e.* in the absence of experimental or model-based initial phase estimates, although it is likely that some weak real-space information is required for successful phasing. We consider here the case where we have knowledge of an approximate (low-resolution) mol­ecular envelope, within which the mol­ecular density is confined, as well as the relative positions of the mol­ecules in the different unit cells, *i.e.* the inter­crystal position operators 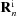 and 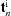. Note that the space-group operators of the crystals will be known from the measured diffraction patterns.

The problem considered is related to that of reconstructing an object from measurements of diffraction from clusters of the object, recently considered by Chen *et al.* (2016 ▸). They consider the case where the diffraction is incoherently averaged over the clusters, and considered clusters that are not crystalline so that the diffraction is measurable continuously in Fourier (reciprocal) space. In the case considered here, the unit cell of each crystal form can be considered a different cluster, *i.e.* a cluster of the corresponding mol­ecules or asymmetric units, and because measurements are made from each crystal form, we have available the diffraction from each cluster, as opposed to only the diffraction averaged over all clusters in the case of Chen *et al.* (2016 ▸). However, in the case considered here, the diffraction is available only at the Bragg positions, as a result of the crystalline nature of the specimens (clusters), as opposed to being available continuously in reciprocal space. Therefore, the problem considered here is on the one hand easier since the data are not averaged, but is on the other more difficult because the diffraction intensities are Bragg sampled.

Chen *et al.* (2016 ▸) describe two approaches, that they call approach A and approach B, for reconstruction of an object from intensities averaged over a number of object clusters. These same two kinds of approaches can be applied to the problem at hand. Approach B has some advantages over approach A, but it is informative to consider both approaches for our problem.

The primary difference between the two approaches, in the context of our problem, is as follows. Approach A explicitly reconstructs the unit-cell contents of each crystal, and then extracts an estimate of the mol­ecule from these. Approach B, on the other hand, reconstructs the mol­ecule in each asymmetric unit by itself (*i.e.* not associated with the other asymmetric units in the crystal), and these are used to form an estimate of the mol­ecule. An important difference between the two approaches is the way that overlap between the mol­ecular envelopes is treated. Although the mol­ecules themselves do not overlap, it is likely that their low-resolution envelopes do overlap within the unit cell. With approach A, overlap introduces errors when the mol­ecule is extracted from the unit cells, since the overlap regions are also extracted. This is avoided in approach B since the mol­ecules (asymmetric units) are reconstructed in isolation.

In the following subsections we describe the constraints and projections for our problem that are used to define an IPA for *ab initio* reconstruction of the mol­ecule.

### 5.1. Constraints   

The data or Fourier amplitude constraint expresses the fact that the Fourier amplitudes of the mol­ecular electron density in crystal *n* correspond to the measured diffraction intensities 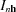, *i.e.* the data constraint can be expressed as
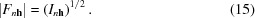
We consider two object or real-space constraints, that we call the support constraint and the similarity constraint.

We suppose that we know the approximate envelope (low-resolution shape) of the mol­ecule, as well as its approximate location relative to the unit-cell origin in crystal 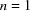. The envelope, also referred to as the support region, is denoted *S* and contains the entire mol­ecule. The density outside the envelope is assumed to be zero (non-zero-valued solvent can be accommodated by not enforcing any particular value on the amplitude of the zero-order reflection). The envelope constraint can then be expressed as
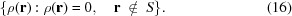
We also denote by 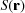 the *indicator function* of *S, i.e.* 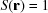 for 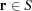 and 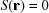 for 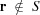.

The similarity constraint expresses the assumption in our model that the mol­ecules in the different crystal forms are identical. We use the term *similarity* rather than *identity* to acknowledge that the mol­ecules in the different crystal forms will generally be slightly different. The similarity constraint can be written as
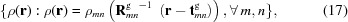

*i.e.* the mol­ecules 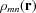 are identical to the reference mol­ecule 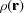 after aligning them back into the position of the reference mol­ecule.

### 5.2. Data projection operator   

Here we derive the projection operator 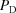 that projects the iterate onto the data, or Fourier amplitude, constraint. As already mentioned above, we define two different approaches A and B, which give different projection operators. A key novelty here is approach B, which circumvents difficulties presented by envelope overlap, as is explained in this and the following subsections.

#### 5.2.1. Approach A   

**Figure 1:**
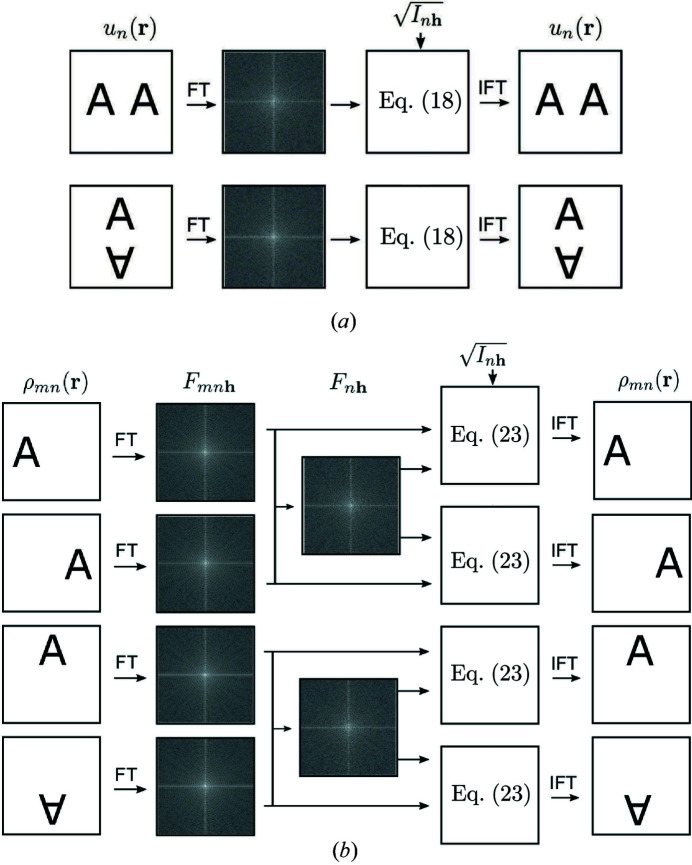
Flow charts showing operation of the data projection for (*a*) approach A and (*b*) approach B.

For approach A, the algorithm operates on the unit-cell densities 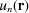. The data projection makes the smallest change to the current estimate of 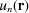 to give a density whose Bragg amplitudes are equal to the measured amplitudes 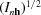. Since the Fourier transform is a unitary linear operator, the projection can be calculated in reciprocal space, treating 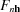 as the iterate. The projection simply projects each complex number 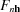 onto the circle of radius 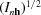 in the complex plane that represents the structure factor. This modifies the amplitude of the current estimate, but leaves the phase unchanged. The projection operator is then given by
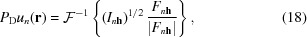
remembering that 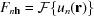, and where 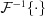 denotes the inverse Fourier transform operation. A flow chart of the operation of the data projection approach A is shown in Fig. 1 ▸(*a*). This projection corresponds to the usual Fourier-space projection for a single object (Millane & Lo, 2013 ▸).

#### 5.2.2. Approach B   

An alternative approach is to define the iterate as the set of separate, rotated and translated mol­ecules 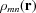, rather than the combined unit-cell densities 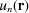. We refer to this as approach B. As is described in Section 5.3, the motivation for this approach is that it accrues an important advantage in eliminating the deleterious effects of envelope overlap that exist with approach A.

As with approach A, we are free to define the projection operators to operate in reciprocal space. We therefore introduce 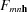 as the transform of the isolated mol­ecule 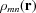, sampled at the reciprocal crystal lattice points, *i.e.* 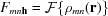, or
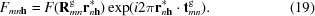
Using this notation, (7) becomes
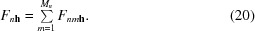
The data projection then involves finding the smallest change to the complex numbers 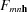, for 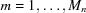, such that 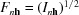. This problem is a special case of approach B described by Chen *et al.* (2016 ▸) and we follow their analysis. Considering a particular crystal *n* and a particular reciprocal-lattice point 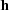, dropping the labels *n* and 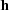, and letting 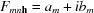, allows (20) to be rewritten as
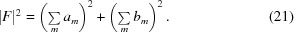
The quantity 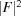 in (21) can be considered to exist in a space of dimension 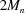, *i.e.* one dimension each for the real (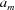) and imaginary (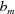) parts of the structure factor for each *m*. Transforming the coordinate system (Chen *et al.*, 2016 ▸) such that two coordinates are defined by 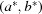, where 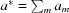 and 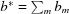, (21) can be written as
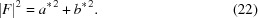
Equation (22) represents a circle in these two coordinates and is independent of the remaining 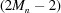 coordinates, in the new coordinate system. Equation (21) therefore represents a 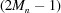-dimensional circular-hyper-cylinder in the 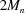-dimensional space representing the iterate 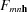 for fixed *n* and 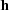. This cylindrical manifold, with the left-hand side of (21) replaced by the measured intensity *I*, is the constraint set representing the diffraction data for a particular crystal at a particular reciprocal-lattice point. The required reciprocal-space projection then moves an arbitrary point in the vector space to the closest point on this cylinder. The projection can be derived using the method of Lagrange multipliers as described by Chen *et al.* (2016 ▸), giving the result
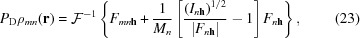
remembering that 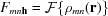 and 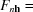 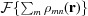. Inspection of (23) shows that the new transform 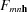 is obtained by taking the mean of the difference between the data and the current individual transforms and adding it to the current value of 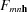. A flow chart of the operation of the data projection approach B is shown in Fig. 1 ▸(*b*). The difference between (23) and (18) corresponds to projecting onto a hyper-cylinder, rather than onto a circle. Equation (23) can be verified by substituting the case of a single cluster with equally weighted objects into the projection derived by Chen *et al.* (2016 ▸).

The data projection operator (23) is in fact a special case of the data projection operator derived by Morgan *et al.* (2019 ▸) for the case of phasing diffraction data from disordered crystals. The two projection operators are formulated somewhat differently however, and the relationship between them is outlined in Appendix *B* [App appb].

### 5.3. Object projection operator   

Here we describe the operator 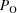 that projects the iterate onto the real-space, or object, constraints described above. The two object constraints are satisfied, in the least-distance sense, by averaging the electron densities over all mol­ecules in all crystals, and applying the support constraint. Again, we consider approaches A and B.

#### 5.3.1. Approach A   

**Figure 2:**
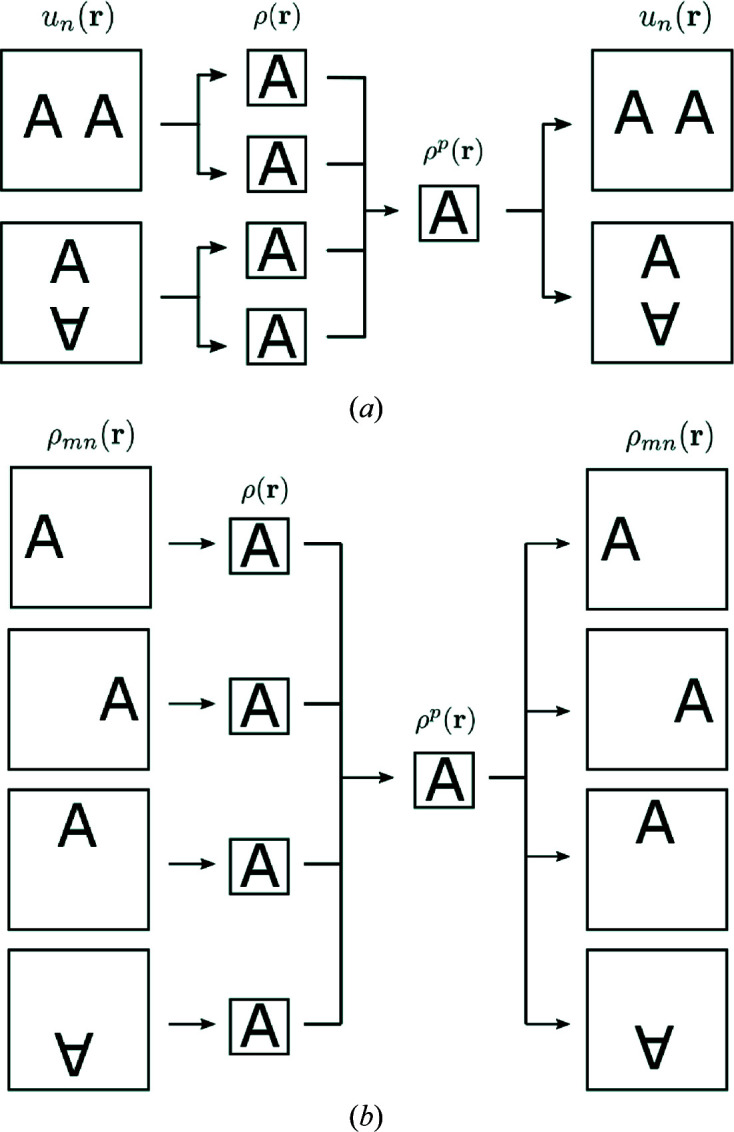
Flow charts showing operation of the object projection for (*a*) approach A and (*b*) approach B.

In approach A, the algorithm operates on the unit-cell density 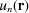. For application of the similarity constraint, each mol­ecule in each crystal is first extracted from the unit-cell density. This is done by applying the support operator, which corresponds to multiplying 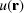 by 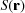, in the appropriate position [step one in equation (24) below]. Each extracted mol­ecule is then aligned (by translation and rotation back to the reference position), followed by averaging to satisfy the similarity constraint (step two). The unit cells are then rebuilt to obtain the new unit-cell densities (step three). The complete object-space projection is then given by
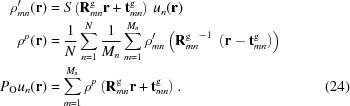
A flow chart of the operation of the object projection approach A is shown in Fig. 2 ▸(*a*).

The necessity of extracting each mol­ecule from the unit cell may lead to problems in tightly packed crystals (which protein crystals usually are). Since the support will always be a little loose, it will generally contain density from neighbouring, symmetry-related mol­ecules, which will contaminate the extracted mol­ecular density. Implementation of the similarity constraint will then be partially incorrect, since the common component will be the density in the support region rather than the density of the mol­ecule. This in turn leads to a defective unit-cell rebuild 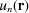 and errors in the updated iterate. Obviously, these errors will be larger, the more the envelopes overlap. Approach B, described below, circumvents this problem.

#### 5.3.2. Approach B   

In approach B, the iterate is the set of all the individual positioned mol­ecules 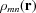, rather than the unit cells. Since the individual mol­ecules are carried through the algorithm, they do not need to be extracted from the unit-cell densities and the issues described above with overlap of the envelopes do not arise. This is the key rationale, and advantage, of approach B. In this case, both object constraints can be satisfied in a single step. The first step in (24) is not needed, and in the third step the averaged mol­ecule needs only to be rotated and translated into its correct position. Thus, the object projection for approach B is given by
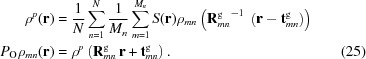
A flow chart of the operation of the object projection approach B is shown in Fig. 2 ▸(*b*).

With all the projection operators now defined, they are easily incorporated into an IPA.

## 6. Simulations   

Simulation results are presented to illustrate the proposed algorithm. The objective is to evaluate the algorithm using synthetic diffraction data that are calculated assuming identical mol­ecules in the different crystal forms. For these tests, we further assume knowledge of a mol­ecular envelope and the inter­crystal position operators. Further tests with more realistic data will be needed to fully evaluate the algorithm’s practical potential.

Two sets of simulations were conducted. The first considers the case of two quite different unit cells with different space groups. Two lysozyme structures were used for these simulations. The second considers the case of multiple unit cells with similar dimensions, as might be obtained in a hydration series, for example. Sets of unit-cell dimensions derived from an X-ray free-electron laser (XFEL) experiment with photosystem II were used for these simulations.

### 6.1. Lysozyme   

**Figure 3:**
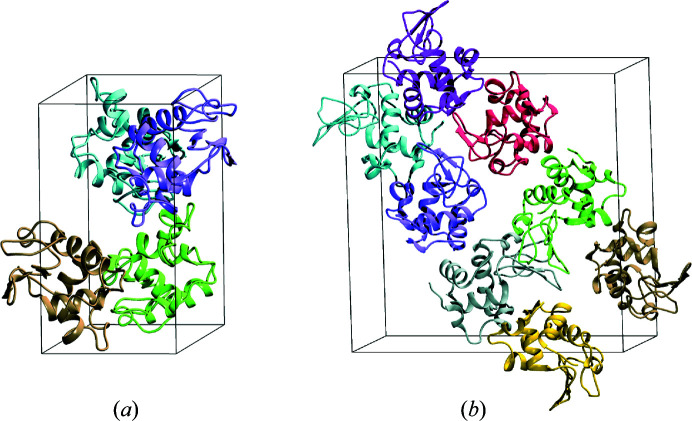
The unit cells for lysozyme in (*a*) crystal form 1 and (*b*) crystal form 2, showing the four and eight mol­ecules in the unit cell, respectively, in the two crystal forms.

For these simulations we use two crystal forms of lysozyme in two different space groups (Fig. 3 ▸). The crystal forms used are Protein Data Bank (PDB) code 132L, which has unit-cell dimensions 30.6 × 56.3 × 73.2 Å and space group 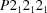, with four mol­ecules in the unit cell (Rypniewski *et al.*, 1993 ▸), and PDB code 193L, with unit-cell dimensions 78.54 × 78.54 × 37.77 Å, space group 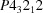 and eight mol­ecules in the unit cell (Vaney *et al.*, 1996 ▸). We refer to these as unit cells 1 and 2, respectively.

Both crystals have fairly low solvent contents (44% and 39%), and thus diffraction data from one crystal form alone are insufficient for *ab initio* phasing, with 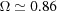. However, with data from two crystal forms and using the current approach, 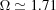 and *ab initio* phasing should be feasible.

An integral number of samples across the unit cells of different dimensions necessitates different sample spacings, and there are different orientations of the mol­ecules with respect to the sampling grids in the two crystals, requiring inter­polation of the electron density. The sampling grids in real space are chosen such that the sample spacings are similar for the two unit cells, and such that averaging over the asymmetric units within the unit cells does not require inter­polation. Unit cell 1 was sampled on a 32 × 60 × 80 grid, giving sample spacings of 0.96 × 0.94 × 0.92 Å, and unit cell 2 was sampled on an 80 × 80 × 40 grid, giving sample spacings of 0.98 × 0.98 × 0.94 Å. Averaging the densities between unit cells, as well as redistributing the average density back into each asymmetric unit in each unit cell to generate the updated 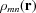, was conducted using cubic inter­polation.

The simulations were set up as follows. Using the corresponding PDB mol­ecular models for each crystal form, a chosen reference asymmetric unit (mol­ecule) in crystal form 1 was aligned with a chosen reference asymmetric unit in crystal form 2, and the inter­crystal position operators determined. The difference between the aligned mol­ecular structures in the two crystal forms is small [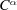 r.m.s.d. (root-mean-square deviation) calculated using *Chimera* (Pettersen *et al.*, 2004 ▸) of 0.83 Å]. Structural differences are concentrated near the periphery of the mol­ecule, while the core structure is well conserved. The reference mol­ecule, or model, from crystal form 1 was placed in crystal form 2 in the position defined by the above-determined inter­crystal position operators, thus generating a crystal structure for crystal form 2 with the identical mol­ecule to that of crystal form 1. Using these models, electron densities (for envelope and real-space error calculations) and structure-factor amplitudes (for the synthetic data) were calculated for each crystal form.

**Figure 4:**
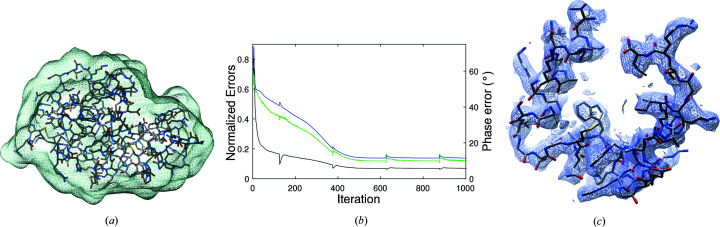
(*a*) The lysozyme 10 Å resolution envelope, with the PDB structure 193L shown for reference, used for the simulations. (*b*) The error metrics *E* (black), Φ (green) and *e* (blue), versus iteration for a typical converged run. (*c*) The corresponding reconstructed electron density associated with amino acids 89–129 contoured at 1.5σ. The electron-density map is displayed using *Chimera* (Pettersen *et al.*, 2004 ▸) with zoning applied to visualize the relevant subregion of the map. The corresponding PDB atomic model of 193L is shown for reference.

An envelope for the (common) mol­ecule was determined by convolving its electron density with a Gaussian with full width at half-maximum (FWHM) of 10 Å and defining a binary mask based on a threshold value of the blurred electron density, giving an envelope resolution of approximately 10 Å, as shown in Fig. 4 ▸(*a*). The solvent fractions of the mol­ecular envelope in unit cells 1 and 2 are 0.33 and 0.24, respectively. These values are smaller than the actual crystal solvent fractions (0.44 and 0.39), and thus the envelope forms reasonably weak *a priori* information. The constraint ratio for the two crystal forms based on these envelopes is 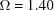. The overlap volumes of the envelope in unit cells 1 and 2 are 0.02 and 0.03 of the envelope volume, respectively.

Phase retrieval was conducted as described above using approach B, with diffraction amplitude data calculated to a resolution of 2 Å. The reconstruction algorithm consisted of repeated cycles of 275 iterations, each cycle consisting of 250 iterations of the DM algorithm with 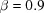, and 25 cycles of the ER algorithm. (Simulations using approach A produced inferior electron-density maps, as a result of the incorrect treatment of envelope overlap.) The algorithm was started with a random electron density within the mol­ecular envelope. In this case, the electron-density iterate consists of 12 copies of the mol­ecule, *i.e.* the 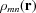. The algorithm was run multiple times with 5000 iterations in each run, and convergence to a good solution was obtained for approximately 50% of the runs. Convergence of the algorithm was monitored by calculating the normalized r.m.s. (root-mean-square) errors for the structure-factor amplitudes (denoted *E*) and the electron density (denoted *e*), and the mean phase error weighted by the corresponding structure-factor amplitude (denoted Φ), all between the current estimate of the electron density and the true values, versus iteration. These error metrics versus iteration for a typical converged run are shown in Fig. 4 ▸(*b*). In this case, the final errors are 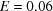, 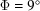 and 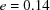. The reconstructed electron density in the region of amino acids 89 through 129 is shown in Fig. 4 ▸(*c*), with the PDB model for reference. The quality of the map is clearly sufficient for chain tracing. Other regions of the map are of similar quality. Although the effects of differences between the mol­ecular structures in the two crystal forms and the effects of errors in the data also need to be considered, the results show the practical potential of the proposed algorithm.

### 6.2. Photosystem II   

The second set of simulations is based on the results of an XFEL experiment with photosystem II (PSII) crystals. Diffraction was measured in a fixed-target, serial femtosecond X-ray crystallography (SFX) experiment at the Linac Coherent Light Source (LCLS) (Metz *et al.*, 2021 ▸). Analysis of the diffraction data from this experiment showed that the crystals exhibited a range of unit-cell constants, due to variation in the relative humidity across the chip on which the crystals were mounted. The unit-cell constants derived from the experimental data are used as the basis for the simulations.

Details of the experiment are reported elsewhere (Metz *et al.*, 2021 ▸), but are outlined briefly here. Crystals of PSII with a size range of 10–50 µm were grown in aqueous solution (Kupitz *et al.*, 2014 ▸). To collect diffraction data, the crystals were spread on a Roadrunner II chip (a porous silicon chip with dimensions of 33 × 12 mm) and the mother liquor around the crystals removed (Tolstikova *et al.*, 2019 ▸). In order to prevent complete dehydration of the crystals, the chips were mounted in a humidity chamber supplied with saturated helium. Due to the relatively large sample holder and the space required for the translation stage, it was difficult to maintain a constant humidity throughout the entire measurement chamber.

As a result of humidity variations across the chip, the crystals showed a range of hydration stages, which was reflected in a continuously varying range of unit-cell dimensions. The space group 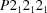 was retained in all crystals. Information on the cell constants observed is reported elsewhere (Metz *et al.*, 2021 ▸), but for our purposes, each diffraction pattern was assigned to one of 14 bins, based on the unit-cell volume, and the data merged within each bin to give 14 diffraction data sets, with a corresponding narrow range of unit-cell dimensions for each (Table 1 ▸). The relative unit-cell volumes are also listed in the table. The largest relative change in the unit-cell dimensions is about 7% for the *a* axis, and the largest change in the unit-cell volume is about 11%.

This set of 14 crystal forms, with similar cell constants and the same space group, was used as the basis for the simulations. The ultimate idea is that data from such an experiment could potentially be used for *ab initio* phasing.

PSII is a large membrane protein complex with a mol­ecular weight of about 350 kDa, with largest dimension of about 120 Å. It exists in nature, as well as in the crystal, as a homodimer, with the two monomers in the dimer related by a twofold symmetry axis (Zouni *et al.*, 2001 ▸). The unit cell contains four copies of the dimeric PSII complex in space group 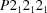, and the twofold relationship between the two monomers of a dimer is non-crystallographic. Although non-crystallographic symmetry (NCS) can be used in phasing, for our purposes we ignore the NCS and treat the whole dimeric complex as the mol­ecule 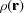 to be reconstructed.

Since the structure of photosystem II is known, the crystal structure for each unit cell was determined by MR as described by Metz *et al.* (2021 ▸). The structures are quite similar and the atomic r.m.s.d.s to the structure in unit cell 1 are listed in Table 2 ▸. The reference electron density 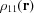 was calculated on a 2.5 Å grid using the atomic coordinates and temperature factors for the structure for unit cell 1, and set to zero outside the mol­ecular envelope (derived as described below). The positions and orientations of the mol­ecules in the 14 unit cells relative to the reference mol­ecule were determined based on the atomic coordinates and using the Kabsch algorithm (Kabsch, 1976 ▸). The rotations were very small and so were set to zero in the subsequent calculations. The reference electron density was then copied into the symmetry-equivalent positions in 14 unit cells, using the derived inter­crystal position operators and Fourier inter­polation. To enable the space-group symmetry operations to be performed without inter­polation, the unit cell constants in Table 1 ▸ were rounded to an integral number of grid points. These electron densities were used to calculate synthetic diffraction data for each crystal form to 5 Å resolution. The lower resolution was used here as a result of the larger unit cell compared with lysozyme, to reduce the computational cost of the reconstructions.

**Figure 5:**
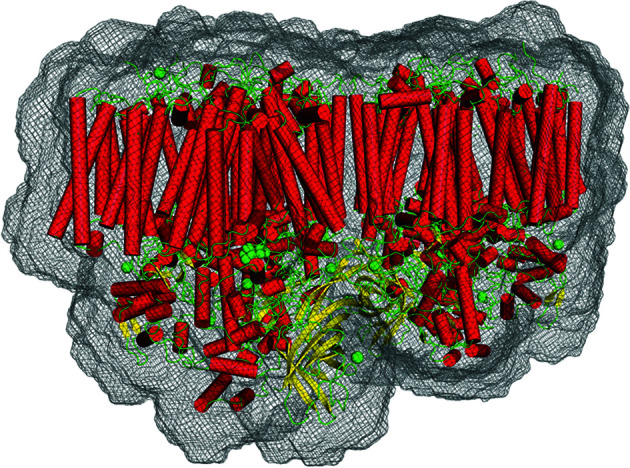
The PSII envelope for 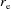 = 8 Å (black mesh), derived as described in the text. The PSII model is shown for reference, with the helices in red, beta sheets in yellow, and loops and metal ions in green. (Figure displayed using *PyMOL* Mol­ecular Graphics System Version 2.4, Schrödinger, Inc.)

In order to study the effect of envelope resolution in this case, the envelope used for PSII was calculated based on the atomic coordinates. The envelope is defined as the union of spheres, of radius 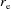, centred on each atom, excluding the hydrogen atoms. The resolution (and the volume) of the envelope can then be easily manipulated by varying 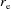. The PSII envelope for 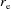 = 8 Å (this can be thought of as an envelope resolution of approximately 16 Å) is shown in Fig. 5 ▸. The envelope volumes relative to the volume of the unit cell for 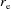 = 8 Å and 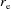 = 7 Å are listed in Table 2 ▸. These quantities decrease with increasing unit-cell size, and increase with increasing 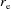, as expected. Since a low-resolution envelope generally includes solvent mol­ecules, the volume fraction of such an envelope will be larger than the protein fraction as calculated by the Matthews coefficient. The protein fraction calculated via the Matthews coefficient is also listed in Table 2 ▸. The envelope fractional volumes are significantly larger than the protein fractions, and thus represent rather weak *a priori* information. The volume of the overlap regions between the envelopes, relative to the envelope volume, is also shown in Table 2 ▸. These also decrease with increasing unit-cell size, and increase with increasing 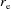. The relative volume of the overlap region is small in all cases.

Using the inter­crystal position operators and mol­ecular envelope as described above, phase retrieval, starting with a random density, was conducted using the DM algorithm and approach B, as for lysozyme. Different subsets of the 14 data sets were used to examine the effect of the number of crystal forms used. Since the algorithm sometimes fluctuated near convergence, a small number of cycles of ER was used at the end of each run.

For a single crystal form with the envelopes used here, 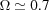 and *ab initio* phasing from such a data set is not expected to be feasible. *Ab initio* phasing was attempted using our approach with data from a single crystal form and, although a small reciprocal-space error was obtained, the resulting density did not resemble the true density, confirming non-uniqueness of the solution in this case.

**Figure 6:**
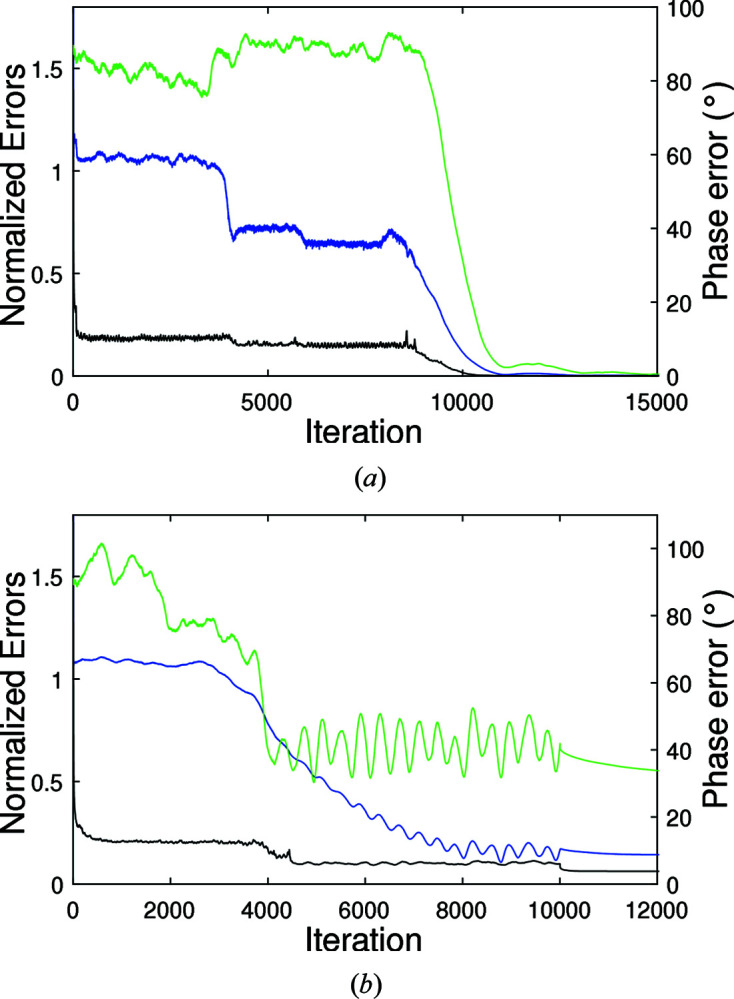
Error metrics *E* (black), Φ (green) and *e* (blue), versus iteration for converged runs of the reconstruction algorithm. (*a*) Using noise-free 5 Å resolution diffraction data from 14 crystal forms with 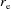 = 8 Å. (*b*) Using noisy diffraction data with a resolution range of 50–5 Å, with data from 14 unit cells and 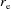 = 7 Å, as described in the text.

**Figure 7:**
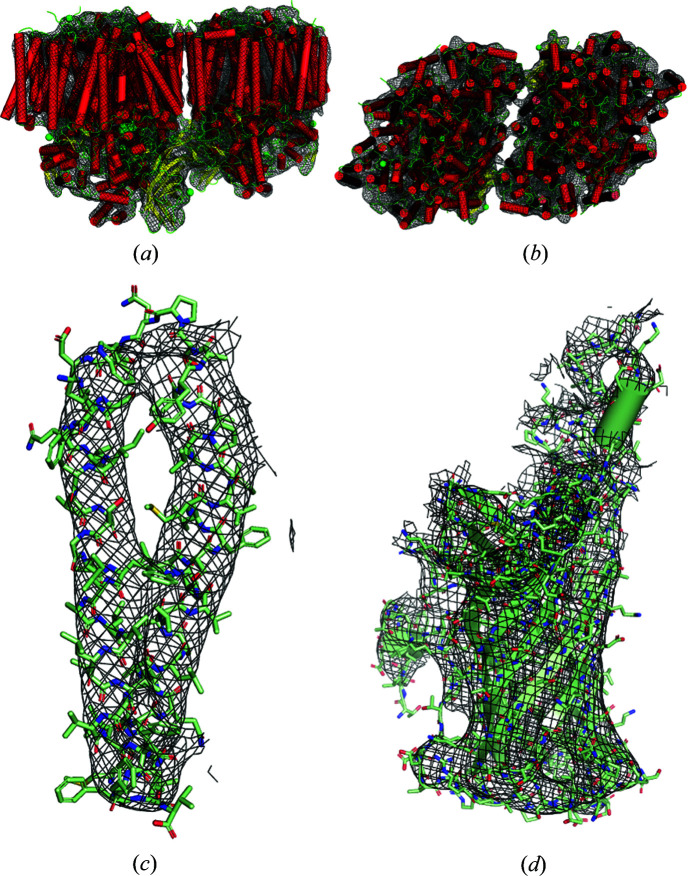
Reconstruction of PSII using noise-free 5 Å resolution diffraction data from 14 crystal forms with 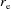 = 8 Å. (*a*), (*b*) Orthogonal views of the electron density of the dimer, (*c*) of chain *z* and (*d*) of the manganese-stabilizing protein, part of the oxygen-evolving complex (black mesh), with the true model shown overlaid. Note that these are 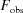 maps with no model refinement. (Figure displayed using *PyMOL* Mol­ecular Graphics System Version 2.4, Schrödinger, Inc.)

The algorithm was run using two, three, eight and 14 of the crystal data sets, for envelopes with 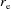 = 8 Å and 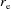 = 7 Å. The results are summarized in Table 3 ▸, which shows the number of converged and total runs in each case. Convergence was defined by a normalized real-space error less than 0.2. As expected, the proportion of runs that converge increases with the number of crystal data sets used and for the tighter envelope (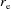 = 7 Å). For the converged runs, acceptable electron densities were obtained in each case. The error metrics versus iteration for a typical converged run are shown in Fig. 6 ▸(*a*), and the corresponding reconstructed electron density in Fig. 7 ▸, for data from 14 unit cells and 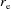 = 8 Å. A good reconstruction at this resolution is evident.

**Figure 8:**
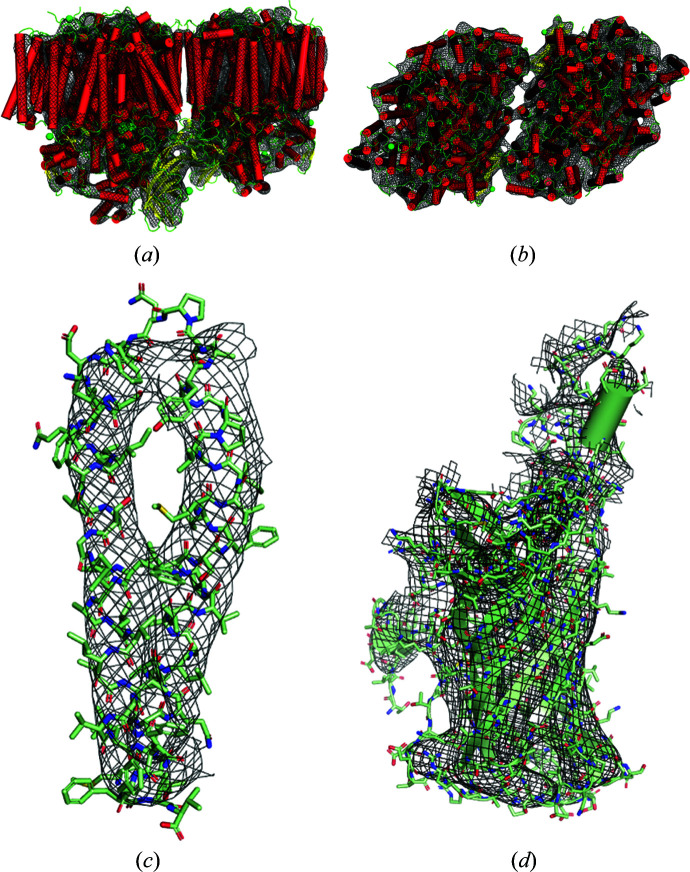
Reconstructions of PSII using noisy diffraction data with a resolution range of 50–5 Å, with data from 14 unit cells and 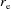 = 7 Å, as described in the text. (*a*), (*b*) Orthogonal views of the electron density of the dimer, (*c*) of chain *z* and (*d*) of the manganese-stabilizing protein (black mesh), with the true model shown overlaid. (Figure displayed using *PyMOL* Mol­ecular Graphics System Version 2.4, Schrödinger, Inc.)

A further, more realistic, set of simulations was conducted in which reflections with resolutions lower than 50 Å and higher than 5 Å were excluded from the data, and errors added to the diffraction amplitudes. Gaussian noise was added to the diffraction data such that 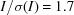 in the highest-resolution shell (5.5–5.0 Å), which represents a reasonably low signal-to-noise ratio (SNR) in practice. In this case, it was found that allowing the excluded structure amplitudes to float (*i.e.* the current amplitude values are retained at each iteration, rather than setting them to zero) in a thin resolution shell at the highest resolution, while decreasing the convergence rate, helped to circumvent the effects of a sharp resolution cutoff. Phase retrieval was successful for three or more crystal data sets and with an envelope with 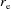 = 7 Å (Table 4 ▸). The quality of the final solution was improved by averaging the maps over multiple converged runs, a common strategy with use of these kinds of algorithms (Morgan *et al.*, 2019 ▸). The error metrics versus iteration are shown in Fig. 6 ▸(*b*), and the reconstructed density in Fig. 8 ▸, for a typical converged run using 14 data sets. Inspection of the figure shows a good-quality reconstruction at this resolution. Overall, the algorithm shows good convergence behaviour in the presence of realistic noise and missing data.

## 7. Discussion and conclusions   

It is not uncommon for different crystal forms to be observed in protein crystallization experiments, without additional experimental effort. Frequently, data from only the best diffracting crystal are used for structure determination, utilizing conventional phasing techniques. For difficult structures however, data from additional crystal forms may be used to advantage to help resolve problems with phasing. Data from multiple crystal forms have been used in various situations, where experimental or MR phases with a single crystal have been insufficient to obtain a solution.

We have considered here the use of diffraction data from multiple crystal forms for potential *ab initio* phasing. Data from three or more crystal forms, or from two crystal forms in favourable cases, with some additional information, such as mol­ecular envelope information, are sufficient to define a unique solution in the absence of initial phase information. An algorithm with good global convergence starting from random phases, to find the solution, is developed using the method of iterated projections. The algorithm incorporates a novel method to circumvent difficulties posed by envelope overlap. Assuming knowledge of a low-resolution mol­ecular envelope and the positional relationships between the mol­ecules in the different crystals, the algorithm is effective in recovering an accurate electron density from simulated data.

Simulations show that our approach has good prospects for an *ab initio* phasing algorithm using data from multiple crystal forms. Our simulations used some simplifications in terms of identical mol­ecules in each crystal form and a well defined protein boundary, and some more work is needed to relax these restrictions. The main additional ingredient required for *ab initio* phasing is a method for determining the mol­ecular envelope and inter­crystal position operators *a priori*. Mol­ecular envelope information may come from another source such as solution scattering or electron microscopy. Even in the absence of experimental envelope information, *a priori* derivation and refinement of an envelope are possible (He & Su, 2015 ▸; Marchesini *et al.*, 2003 ▸). Note also that steric constraints on the envelope will be stronger when considering the different packings in multiple unit cells. Determination of inter­crystal position operators is the same problem as is encountered in mol­ecular replacement and multi-crystal averaging. The inter­crystal rotation operators should be obtainable from a cross-rotation function. Determination of the translation operators is, as usual, more difficult, but X-ray agreement and steric searches can be effective in constraining the possible translations. In summary then, determination of these other parameters to allow *ab initio* phasing would appear to be feasible. Success with this approach would allow ultimately for the practical realization of a phasing method that was first proposed long ago in early work by Perutz and Rossmann.

Improvements will be needed to make the method effective with experimental data. In the simulations used here, a mol­ecular electron density with a sharp cutoff at the envelope boundary was used to calculate the data. In practice, a softer support projection may help to reduce the effect of ripples in the Fourier domain that are introduced by a sharp cutoff in real space. The inter­polation methods used are also important. Real-space inter­polation methods, such as cubic inter­polation, have the disadvantage that they tend to smear the electron density, whereas Fourier inter­polation tends to extend the density beyond the mol­ecular boundary. These effects are important since inter­polation is conducted over many iterations. Fourier inter­polation being inconsistent with the support boundary was circumvented in the simulations by using data sets which were calculated from mol­ecular densities that included the Fourier artefacts. Further investigation of suitable inter­polation methods is needed.

Different crystal forms can occur with routine crystal screening and provide data suitable for the approach described here. Systematic manipulation of parameters such as humidity, salt concentrations *etc*. may also provide a range of crystal packings. Recent SFX experiments with PSII (Metz *et al.*, 2021 ▸) show a potential method for obtaining data from a range of crystal forms.

## Figures and Tables

**Figure 9:**
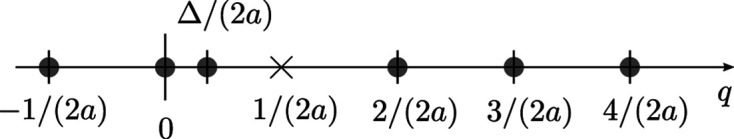
A regular sampling sequence of spacing 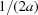, except that one sample has been shifted to a position that is a distance 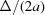 from one of its neighbouring samples, shown by the circles. The original position of the shifted sample is shown by the cross.

**Table 1:** Average unit-cell constants for binned PSII diffraction data and relative unit-cell volumes 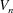
 = unit-cell volume of crystal form *n*.

*n*	*a* (Å)	*b* (Å)	*c* (Å)	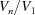
1	130.7	229.7	305.0	1.00
2	131.7	230.1	305.3	1.01
3	132.3	230.2	305.6	1.02
4	132.8	230.3	305.8	1.02
5	133.2	230.3	306.0	1.03
6	133.6	230.4	306.3	1.03
7	134.1	230.5	306.6	1.04
8	134.7	230.8	307.0	1.04
9	135.4	231.0	307.4	1.05
10	136.2	231.3	307.8	1.06
11	137.2	231.6	308.3	1.07
12	138.2	232.0	308.9	1.08
13	139.3	232.4	309.8	1.10
14	140.4	233.0	310.9	1.11

**Table 2:** Atomic r.m.s.d., protein fraction, and relative envelope volumes and relative envelope overlap volumes for two values of 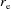, for the PSII unit cells, as described in the text 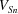
 = envelope volume in unit cell *n*, 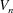 = volume of unit cell *n*, 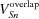 = envelope overlap volume in unit cell *n*.

			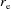 = 7 Å		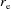 = 8 Å	
*n*	R.m.s.d. (Å)	Protein fraction	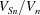	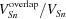	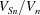	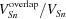
1	0.00	0.37	0.70	0.05	0.74	0.07
2	0.08	0.37	0.68	0.03	0.72	0.05
3	0.12	0.37	0.68	0.03	0.72	0.05
4	0.13	0.36	0.68	0.03	0.72	0.05
5	0.13	0.36	0.68	0.03	0.72	0.05
6	0.14	0.36	0.68	0.03	0.72	0.05
7	0.15	0.36	0.68	0.03	0.72	0.05
8	0.17	0.36	0.68	0.03	0.72	0.05
9	0.19	0.35	0.66	0.03	0.70	0.04
10	0.19	0.35	0.66	0.03	0.70	0.04
11	0.22	0.35	0.66	0.03	0.70	0.04
12	0.23	0.34	0.66	0.03	0.70	0.04
13	0.27	0.34	0.66	0.03	0.70	0.04
14	0.28	0.33	0.63	0.02	0.67	0.03

**Table 3:** Number of converged (‘conv’) and total runs (‘runs’) for different numbers of data sets (*N*), with noise-free diffraction data and envelope radii (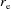) for the simulations with PSII

*N*	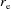 = 8 Å Conv/runs	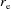 = 7 Å Conv/runs
2	0/150	13/150
3	7/250	113/150
8	42/100	92/100
14	52/100	100/100

**Table 4:** Number of converged (‘conv’) and total runs (‘runs’), and final error metrics, for different numbers of data sets (*N*), with noisy diffraction data and envelope radius 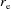 = 7 Å for the simulations with PSII

*N*	Conv/runs	*E*	Φ (°)	*e*
2	0/300	-	-	-
3	25/300	0.12	20.2	0.17
8	96/200	0.17	24.1	0.08
14	88/200	0.18	23.8	0.09

## Appendix A Noise amplification due to a shifted sample

Ideally, the samples of the continuous transform intensity are distributed evenly in reciprocal space. However, as described in Section 3, it is likely that with data from multiple crystal forms, at least some samples will be close together. Here we consider the effect of close proximity of samples of the diffracted amplitude.

The effect on signal reconstruction of a finite number of the Nyquist samples being shifted to new positions has been considered by Yen (1956 ▸). He showed that such a sampling sequence allows unique reconstruction of the signal, and derived the corresponding inter­polation functions. Using his results, we consider the effect of two sampling points being close together.

Following Section 3, consider a 1D real-space lattice of spacing *a*, so that the Nyquist spacing for the Fourier intensity is , although the intensity data from a single crystal form are spaced by only . Consider now a regular sampling sequence with spacing , except that one sample is shifted to a distance  from its neighbouring sample, as considered in Section 3. Let the shifted sample be indexed  and shifted to the left so that its new position is , and it is thus a distance  from the sample  at the origin  (Fig. 9 ▸). The effect of shifting a finite number of samples is that the sampling functions for samples close to a resulting bunch of samples take on large values at positions *q* that are close to the resulting gaps between the samples. This results in noise amplification close to the gaps, the amplification being larger for larger sample shifts. For our case, the sample positions at the bunch are at . Since we wish to reconstruct the signal at the original, regular sample position  (which is in the resulting gap), and since the inter­polation functions are unity at the sampling points, the noise amplification factor is equal to the magnitude of the inter­polation function at , and we denote this value by , for , as a function of Δ. Evaluation of the inter­polation functions given by Yen (1956 ▸) for this case shows that the noise amplification factors for samples in the bunch are given by

Hence, for small Δ, where the effect of the bunching is more significant, the noise is amplified by a factor . For example, if say 5% of the samples were a fractional distance 0.1 (*i.e.* ) from another sample, the noise amplification in the reconstructed electron density would be about 50%.

## Appendix B Alternative formulation of (23)

As mentioned in Section 5.2.2, the data projection operator for approach B (23) can be formulated in an alternative fashion that makes an interesting connection to the data projection operator for the case of a reconstruction problem for disordered crystals described by Morgan *et al.* (2019 ▸). This formulation of our projection operator is derived here.

Consider the Bragg-sampled Fourier transform of the iterate, , but now first take the discrete Fourier transform (DFT) of  with respect to *m*, which we denote , *i.e.*

where  denotes the 1D DFT operation with respect to *m*, and *k* is the variable conjugate to *m*. We now treat  as the iterate, and note, referring to (27), that for ,

so that the data constraint becomes . Letting  be the projection operator acting on the iterate , the projection is then given by

The data projection operator , which acts on the iterate  in real space, is then given by

Equation (30) is then equivalent to (23).

Geometrically, the effect of taking the DFT of  with respect to *m* is to rotate the vector representing the electron density (the iterate) such that the circular cross section of the hyper-cylinder lies in the complex plane of the  component of . The projection then involves simply rescaling the magnitude of  to the data and leaving the other components unchanged, followed by rotating back to the original coordinate system (by taking the inverse DFT). Simplification of the projection operator by rotating the coordinate system via the DFT is identical to the approach used by Morgan *et al.* (2019 ▸) to simplify the projection operator in the case of phase retrieval for diffraction data from disordered crystals.

The equivalence of (23) and (30) can be shown directly as follows. Referring to equations (27), (28) and (29), we can write the projection operator in reciprocal space as

where  is the Kronecker delta function. Substituting (31) into equation (30) and writing out the inverse DFT gives

which is (23).
